# Simple methods to obtain food listing and portion size distribution estimates for use in semi-quantitative dietary assessment methods

**DOI:** 10.1371/journal.pone.0217379

**Published:** 2019-10-18

**Authors:** Christine Hotz, Lubowa Abdelrahman

**Affiliations:** Global Alliance for Improved Nutrition (GAIN), Geneva, Switzerland; McMaster University, CANADA

## Abstract

Semi-quantitative dietary assessment methods are frequently used in low income countries, and the use of photographic series for portion size estimation is gaining popularity. However, when adequate data on commonly consumed foods and portion sizes are not available to design these tools, alternative data sources are needed. This study aimed to develop and test methods to: (i) identify foods likely to be consumed in a study population in rural Uganda, and; (ii) to derive distributions of portion sizes for common foods and dishes. A process was designed to derive detailed food and recipe lists using guided group interviews with women from the survey population, including a score for the likelihood of foods being consumed. A rapid recall method for portion size distribution estimation (PSDE) using direct weight by a representative sample of the survey population was designed and implemented. Results were compared to data from a 24 hour dietary recall (24HR). Of the 82 food items reported in the 24HR survey, 87% were among those scored with a high or medium likelihood of being consumed and accounted for 95% of kilocalories. Of the most frequently reported foods in the 24HR, portion sizes for many (15/25), but not all foods did not differ significantly (p<0.05) from those in the portion size estimation method. The percent of portion sizes reported in the 24 hour recall falling between the 5th and 95th percentiles as determined by the PSDE method ranged from 18% up to 100%. In conclusion, a simple food listing and scoring method effectively identified foods most likely to occur in a dietary survey. A novel PSDE method produced similar estimates as for the 24HR, while the approach for others should be further considered and validated. These methods are an improvement on those in current use.

## Introduction

Dietary assessment surveys are necessary to adequately inform, design and evaluate nutrition intervention programs in low-income countries. While the 24-hour dietary recall (24HR) is one of the most common methods used in these settings [1], it is also more resource intensive and technically challenging than other methods [2,3] and this may be a limiting factor for the use of dietary surveys to inform effective nutrition programs. Depending on the specific objectives of a dietary survey, simpler and less resource intensive methods, including food frequency questionnaires (FFQ) or semi-quantitative (SQ) FFQ, may be adequate [4]. The semi-quantitative estimation of portion sizes consumed using food photo series or atlases depicting graduated portion sizes for a variety of foods is gaining popularity, and may be used to support the application of SQ-FFQs [5] or as a way of simplifying 24HR methods [6,7].

However, to adequately develop such SQ dietary assessment tools, some key information is required beforehand. This includes, but is not limited to: (i) a listing of the foods that are commonly consumed in the study population and hence should be included in the SQ tools; (ii) relevant details about the way they are typically prepared or consumed, and; (iii) the distribution of usual portion sizes to select those to represent in the SQ tools. Ideally, data-driven methods in the form of previously collected dietary intake survey data that is quantitative, valid, and representing the same survey population and sub-population groups of interest, would serve this purpose [5,8]. In low-income countries where data meeting these criteria may often not be available, some form of reliable, empirically-derived preliminary data are needed.

We have found few well-described or well-designed processes in the published literature on how to collect food listing and portion size distribution data when appropriate previous survey data are not available. Food listings have been derived using informal or subjective methods such as consultation with food service professionals, local cook books, or restaurant and cafeteria menus, or conducting interviews with cooks or chefs in households and restaurants, but without any information on the sample size, sampling frame or representativeness [6,9,10]. While these are relatively low-cost methods, it is not clear how complete or representative they are.

In the absence of pre-existing data, portion size ranges have been deduced by various means, such as by adapting from local reference data (e.g., dietary guidelines and nationally established standard serving sizes), consulting experts in the catering industry, qualitative consultation with households [9–11], or deriving a medium portion size from existing survey data but applying fixed ratios to derive small and large portion sizes [9,12]. In some studies to develop and validate food photo atlases, either very scant or no information was provided on how graduated portion sizes were derived [10,13,14], confirming that this methodological step is often overlooked. This is concerning as, if portion size options presented in SQ-FFQ or SQ-24HR dietary assessment surveys do not represent the usual range consumed in the population studied, large portion size estimation errors can result [15].

In some studies, mean portion sizes were determined in small surveys where householders were asked to demonstrate usual portion sizes for different foods for specific age groups [6,12,16], but a minimal description of the methods used for sampling or data collection was provided. This may be an innovative way to collect portion size data for dietary survey tool design, but well-described methods using a systematic and representative approach are needed.

The aim of this study was to develop, document and field test data collection methods to determine food listings and portion size distributions for application in dietary assessment studies. We chose to conduct this work among women in rural Uganda, where researchers have experience in conducting large-scale 24HR dietary recall and SQ-FFQ surveys. The main objectives were to develop, document, and field test methods to: (i) create listings of foods likely to be consumed in a study population, and; (ii) to quantitatively derive distributions of portion sizes for commonly consumed foods and composite dishes. Limited comparisons to the frequency of foods reported and estimated portion sizes derived from a 24HR survey conducted in the same population were also made.

## Materials and methods

This study was conducted as part of a larger study to compare dietary intake outcomes of a SQ 24HR survey method and a SQ-FFQ method with a standard 24HR method (S1 File). The study was conducted in Nakisunga sub-county (population >48,000) in Mukono District, Uganda, a site that was purposively selected for its proximity to Kampala, having a largely rural agricultural livelihood with some urban influence, socio-cultural homogeneity, and the cooperation of local authorities. This study was reviewed and approved by the Higher Degrees, Research Ethics Committee, Makerere University School of Health Sciences, Kampala, Uganda and registered and approved by the Uganda National Council of Science and Technology. Informed written consent was obtained from all participants.

### Study participants and sampling

The sampling frame established for the larger study was used to draw sub-groups of participants for the data collection methods described here. The study included n = 336 women 18–49 years of age, who were residents of the home visited, self-identified as the primary or most senior female caretaker in the household with responsibility for meal preparation, and were available and consented to participate (S1 File). Women who self-reported to be currently pregnant or lactating with a child <23 months of age were excluded to ensure a more homogenous subgroup with regard to intakes and nutritional requirements, which was relevant to the larger study.

We used a multi-stage sampling procedure whereby four of eight parishes in Nakisunga sub-county were randomly selected. Three enumeration areas (EAs), defined by the 2014 Uganda Population and Housing Census sampling frame, were randomly selected from each selected parish. A census of these EAs identified households with eligible women. After dividing the total sample equally among the four parishes, population proportionate sampling of eligible women was done from the three selected EAs per parish for each of the data collection activities.

### Data collection

#### Socioeconomic data

A brief socioeconomic questionnaire was administered to all participants of the larger study. We used the Probability of Poverty Index® (PPI), as validated in Uganda, to compare poverty risk among participants in different data collection activities (http://www.progressoutofpoverty.org/country/uganda). The index provides a simple household level probability score whereby 0 represents the highest probability of poverty and 100 the lowest probability.

#### Food listing

The food listing activity used semi-structured, guided group interviews to: (i) create a list of all foods and beverages commonly consumed by the survey population, including nutritionally-relevant details such as state, processing method, or cooking method; (ii) obtain a categorized, scored ranking of the likelihood of foods being consumed by adult women in the area at the time of the prospective dietary survey (July 2017) with scores of: '1', high; '2', moderate; '3', low; and '4', not likely at all, and; (iii) capture main and optional ingredients of commonly consumed recipes. Two types of interviews were conducted: one with pairs of key informants (KI) and the other with groups of survey participants. Prior to the interviews, project staff created a spread sheet with food group categories and an initial list of foods likely to appear.

Two KI interviews were held, one with district level and one with sub-county level government staff, each including a government agriculture officer and a health officer, selected for their knowledge of the availability of local foods, diets, and seasonal availability. The KI interviews were done to create a locally relevant, initial listing of foods likely to be encountered in the area to inform the structure and content of the interview guide for the survey participant interviews, and also allowed a comparison of the likelihood of consumption scores between the KI and participant interviews. A field coordinator and field staff member conducted the interview using the initial food group/food item list as a guide and as a prompt list for foods not mentioned by the KIs, while another field staff member recorded the information. The purpose of the interview, the information of interest, and the likelihood of consumption scores were explained. Detailed recipe data were not obtained in the KI interviews. Data collected from the two KI interviews were transcribed into a spreadsheet format, details combined, and the mean likelihood scores obtained for each food in the two interviews were calculated, always rounding to the higher likelihood score if they did not conform. This listing was then used to guide the group interviews.

Four interviews were held with one group of 8–10 women from each of 4 parishes. The food groups were divided into two sets, whereby each set was covered in two group interviews that lasted approximately 2.5–3 hours. A structured guide was used to elicit food items consumed, including specific details on the food type (e.g., local name(s), color, variety, commercial products), processing and preparation methods (e.g., whole or milled; mashed or chopped and boiled, steamed, fried, etc.), the likelihood of the food being consumed in the household during the survey period (i.e., high, medium, low, not likely at all), and recipes for mixed dishes prepared. For each mixed dish recipe type mentioned, additional details were obtained, including preparation method, whether ingredients were major or minor components, and a likelihood score for their inclusion in the recipe (i.e., '1', always; '2', often; or '3', rare; this information is used to correctly identify recipes and for the purpose of collecting standard recipe data, a process not reported on here). Finally, a listing of the most common ingredient combinations was obtained. As there are many variations of a recipe and optional ingredients, this listing was done to clarify which of the ingredients mentioned were more commonly combined together in a recipe. The data collection tools used to record and summarize responses for food items/ingredients and for mixed dish recipes, with sample data included, are given in S1 and S2 Figs, respectively.

As for the KIs, information from the two interviews on the same food groups was combined in a spreadsheet (one for food items and one for recipe data), and an average likelihood score of foods or ingredients was obtained, rounding to the higher score as needed.

#### Selection of foods for portion size distribution estimation (PSDE)

Foods that were scored with a high ('1') or medium ('2') likelihood of being consumed in the participant group interviews were reviewed for inclusion in the PSDE method. (n = 43), plus mixed dishes made with those foods (n = 24). For the selection of foods consumed as stand-alone items, n = 43 were scored with a high or medium likelihood of consumption. Of these, 10 were dropped, as one was not found in the market (i.e., apples), one was better estimated as a count than portion size distribution in grams (i.e., hard candies), and 8 were similar to other items or represented variants of the same food type and the portion size was not expected to differ between them (i.e., pork/beef, and different varieties of sweet potato, amaranth leaves, yams and some bananas). To this list, 3 foods that had oil-fried versions and were distinguished as separate food items (e.g., French fried potatoes were listed separately from boiled potatoes) were added, plus 7 processed baked goods items that were not well addressed in the interviews but added by the researchers as they were considered common in the area. Food items that were scored with a low likelihood of consumption ('3') or as unlikely to be consumed ('4') were excluded as they were hypothesized to contribute minimally to population-level energy and nutrient intakes.

For the mixed dishes, 30 common ones had been identified, but 6 were dropped as portion sizes were expected to be the same for very similar mixed dishes. For some mixed dishes for which primary ingredients are substitutable, a mixed dish ‘type’ was used to represent the variations (e.g., dishes made with similar types of green leaves, or with different varieties of common beans were grouped together). A total of 67 stand-alone food items (n = 43) or mixed dishes (n = 24) were included in the PSDE data collection.

#### PSDE method

The PSDE method used a quantitative recall approach with interactive interviews. The 67 selected foods were divided into 4 sets of 16–17 items each. Four subgroups of 56 women (n = 224) invited to participate were asked to recall portion sizes for one of the four sets of foods. We calculated sample sizes for a range of different foods using existing portion size data (grams per serving) from a dietary survey conducted in central and eastern Uganda using the equation: [Zα/2. δ / E]^2^, where Zα/2 = 1.96 = 95% confidence, δ = known SD and E = acceptable error in measurement units. The error (E) was set at the equivalent of a coefficient of variation of 15%. This resulted in sample sizes ranging from n = 13 to 135, and 80% of the 15 sample sizes calculated were <60. We rationalized that portion sizes reported by n = 56 different respondents would be adequate for most foods.

The PSDE sessions were organized in a central location of each parish. All foods and dishes were prepared by locally hired assistants in the form typically served. The sets of 16–17 foods were divided into 3 separate data collection stations, with one interviewer and one person weighing and recording the portion sizes, with each woman completing data collection at one station before moving to the next one. For each food item, the interviewer prompted the woman to recall if she consumed it on the previous day, week, or months. If they could not recall the last time they ate that food, or they never eat that food, no information was collected. If they could recall the last time they ate that food, they were asked to estimate the amount consumed. The respondent was then asked to serve up that amount of food from the real foods provided. These amounts were weighed to the nearest gram on a digital dietary scale, recorded, and the weight of the dish subtracted.

Where portion size data were collected with inedible fractions included (e.g., bones in fish, peel and seeds in watermelon), this was also recorded. Edible fractions for those foods were determined separately by weighing a sample of food items, removing inedible fractions, and then weighing the yield of edible amount on dietary scales. Edible yield factors were then applied to the portion sizes recorded to calculate the weight of the edible portion size.

#### 24HR survey

We used a multiple pass approach based on Gibson and Ferguson [17] with specific methods that were previously described in detail [18]. Participant group 'training' sessions were held in each EA two days before the 24HR interview to explain the purpose of the study, and the methods involved. They were asked to use their own dishes for serving and eating their food the next day to improve visual memory, and instructed on the use of picture charts to mark foods consumed.

Portion sizes of items consumed were estimated using methods specified for each food type. These included life-size graduated photographs, weighing scales, graduated measuring cylinders and play dough models, or standard weights for foods that are served as units (e.g., boiled egg, bread slice) [17]. Portion sizes recorded accounted for any leftovers that were served but not consumed. If multiple servings of the same food item were reported to be consumed in a single eating occasion (e.g., morning, afternoon, or evening meals or snacks) these amounts were combined to a single portion. All of these proxy measures were later converted to gram weights of the food represented using a set of conversion factors.

### Data management and analysis

The CSDietary program (HarvestPlus/Serpro, 2009), using the CSPro software platform (Serpro, Santiago, Chile), was used for dietary data entry and data processing. All data were entered in duplicate and discrepancies were identified and rectified, and distributions of intakes were reviewed for plausibility by examining high and low intakes. All subsequent data management, processing, and analyses were done using Excel (Microsoft Office 2007 for Windows) and SPSS 16.0 and 18.0 for Windows (SPSS Inc., Chicago, IL, USA). For the food listing, the number of foods by likelihood score was determined. For the PSDE data, and portion sizes derived from the 24HR survey, descriptive statistics (mean, SD, CV, and 5th, 50th, and 95th percentiles) were calculated in grams.

To determine whether the distribution of portion sizes derived from the PSDE method were adequate to capture those reported in the 24HR survey, we: (i) calculated the number (percent) of portions reported in the 24HR survey whose gram weights fell within the 5th and 95th percentiles of weight derived in the PSDE activity. These percentiles are suggested to represent the smallest and largest portion sizes in food photo series [8], and; (ii) compared the distributions using the Mann-Whitney U-Test/Wilcoxan Rank Sum Test for two independent samples with unequal sample sizes drawn from the same population, where p<0.05 indicates a statistically significant difference. For the socio-demographic data, each individual indicator or score and the final PPI score was compared between the PSDE method group and the 24HR survey group.

## Results

### Sample and socio-demographic data

Response rates for participants were 78% in the PSDE activity (i.e., n = 214 participants / 274 invited) and 73% in the 24HR survey (i.e., n = 115 participants / 158 invited). Socio-demographic data were derived for only a subset of 86% (184/214) of the PSDE participants as these data were collected only for those who also participated in a larger study, including the 24HR survey presented here (Table 1). Results for these subgroups suggest that they were similar in socio-demographic characteristics. Of the 184 PSDE participants, 57 participated in both the PSDE and the 24HR survey and the data were retained in both groups for this analysis.

**Table 1:** Socio-demographic data for subgroups of participants in the PSDE and 24HR surveys^a^.

		PSDE method^a^		24HR survey^a^		
Characteristic	Response	Mean	SD	Mean	SD	P^b^
n		184^c^		115		
Age (years)		33.6	9.0	33.4	9.0	ns
Number of household members (n)		5.9	2.5	5.7	2.5	ns
		%	CI	%	CI	
All household members own at least one pair of shoes (%)	Yes	81.0	75.3–86.7	76.5	64.7–81.3	ns
All children 6–12 years in school (%)	Yes	67.9	61.2–74.6	64.3	55.5–73.1	ns
	No children 6–12 years	4.9	1.8–8.0	0.9	0.0–2.6	ns
Lead female able to read/write (%)	Yes	77.2	71.1–83.3	79.1	71.7–86.5	ns
Main wall material (%)^d^	Brick, earth or clay	92.4	88.6–96.2	91.3	86.1–96.5	ns
Main roof material (%)^d^	Iron sheets	97.8	95.7–99.9	96.5	93.1–99.9	ns
Toilet facility type (%)^d^	Pit latrine with cement slab	58.7	51.6–65.8	54.8	45.7–63.0	ns
	Pit latrine—no cement slab	20.1	14.3–25.9	23.5	15.8–31.2	
Cooking fuel type (%)^d^	Wood / dung / grass	58.2	51.0–65.3	55.7	46.6–64.8	ns
	Coal	41.8	44.7–49.0	44.3	35.2–53.4	
Number of cell phones (%)	0	7.6	3.8–11.4	2.6	0–5.5	ns
	1	22.3	16.3–28.3	21.7	14.2–29.2	
	2	44.0	36.8–51.2	47.8	38.7–56.9	
	≥3	26.1	19.7–32.3	27.8	19.6–36.0	
PPI score^a^		53.6		54.7		

^a^PSDE, Portion size distribution estimation method; 24HR, 24 hour dietary recall; PPI, Progress out of Poverty.

^b^ANOVA test where means are presented and by Chi-square test where data are categorical; *, P<0.05; ns, non-significant (P≥0.05).

^c^Data on socio-demographic data are available for only 184 of the 214 participants in the PSDE data collection as this questionnaire was only applied to those who participated in a household survey including three dietary assessment methods. Of the 184 PSDE participants, 57 also participated in the 24HR survey presented here and the data were retained in both groups for this analysis.

^d^Data only shown for the primary responses recorded; statistical tests included all possible responses.

### Food listing

The food and recipe listing process identified 77 unique foods (i.e., those consumed as individual food items and those used as ingredients in composite dishes) that were scored by survey participants with a high ('1') or medium ('2') likelihood of being consumed during the survey, including 3 foods with two distinct preparation methods. Likewise, 39 were scored with a low likelihood ('3'), and 57 as not at all likely ('4') or not consumed at all.

Of the 82 distinct foods and ingredients mentioned in the 24HR survey, 71 (87%) were among those scored with a high or medium likelihood of being consumed and accounted for 95% of estimated kilocalorie intake, while 7% were among those scored with a low likelihood or not at all likely, accounting for <1% of estimated kilocalories; of the latter, 5 foods were reported by a single individual and 1 food was reported by 2 individuals. The remaining 6% of foods reported in the 24HR did not appear in the food listing at any stage, and accounted for 5% of estimated kilocalorie intake; of these, sugarcane was reportedly consumed by 26 individuals, while the 4 other foods were reported by ≤4 individuals.

The KI food listing tended to result in higher likelihood of consumption scores of foods than the listing made by survey participants. For example, there were 24 foods scored as not likely or never consumed by the participants that were scored with a high (n = 1), medium (n = 8) or low (n = 15) likelihood of being consumed in the KIs. There were also 10 foods listed as being consumed by the KIs but not mentioned or scored during the group survey participant interviews. The latter were largely comprised of uncommon bean varieties and non-indigenous ('imported') vegetables.

### PSDE method

Descriptive data are presented for the distributions of estimated portion sizes for a selection of individual food items and mixed dishes representing those reported with highest frequency (i.e., >10 occurrences; Table 2) and with low frequency (i.e., 4–6 occurrences; Table 3) in the 24HR survey. The SDs for these food items were relatively large, and the coefficient of variation (CV) for these estimates ranged from 0.27 to 0.98, with an average of 0.47. Portion sizes for approximately half of the individual food items and mixed dishes did not follow a normal distribution (p<0.05).

**Table 2:** Portion sizes (grams) for foods and composite dishes estimated from a portion size recall survey and reported with frequency ≥10% of all food portions in a 24HR survey in the same population^a^.

	PSDE Method							24HR Survey					
Food or Beverage	n	Mean	SD	CV	5th pct	Median	95th pct	n	Mean	SD	CV	Median	Portions within 5^th^-95^th^ pct (%)^b^
		grams							grams				
Individual food items													
Plantain, cooked	54	361	140	0.39	111	369	624	48	485	238	0.49	474	69*
Maize on cob, cooked	53	165	83	0.50	36	139	366	43	163	85	0.52	129	98
Cassava, boiled	50	179	87	0.49	63	179	341	42	225	133	0.59	197	76
Avocado	40	77	39	0.51	23	68	169	42	77	36	0.47	89	95
Mango	43	203	91	0.45	94	180	386	34	98	73	0.74	82	18*
Sweet potato, yellow or white, cooked	49	327	162	0.50	102	327	655	56	322	198	0.61	283	86
Bread, white	58	78	33	0.42	25	78	141	28	160	94	0.59	125	57*
Chapatti	50	129	50	0.39	77	111	252	22	93	37	0.40	94	68*
Banana, large-type	53	113	70	0.62	45	120	215	20	117	66	0.56	104	90
Mandazi (fritters)	47	98	95	0.97	41	91	132	18	63	37	0.59	63	67*
Beef, cooked	53	50	28	0.56	20	43	101	15	102	64	0.63	99	53*
Jackfruit	56	305	174	0.57	124	295	733	13	316	190	0.60	276	100
Pumpkin, cooked	53	231	142	0.61	54	212	541	13	197	63	0.32	190	100
Egg, fried	47	57	28	0.49	27	47	107	11	91	33	0.36	99	82
Mixed dishes													
Bean sauce	51	236	73	0.31	122	220	372	76	191	102	0.53	187	66*
Maize posho (stiff porridge)	49	328	142	0.43	98	340	549	52	329	127	0.39	326	94
Milk tea	55	430	115	0.27	250	398	579	47	422	152	0.36	390	89
Rice dish	49	285	104	0.36	88	301	463	43	297	171	0.58	245	79
Small fish sauce	53	50	20	0.40	19	47	87	24	133	59	0.44	136	21*
Fresh fish soup (broth only)	46	72	38	0.53	29	64	163	19	162	86	0.53	146	58*
Cassava and beans (Katogo)	49	458	163	0.36	177	436	730	17	419	232	0.55	367	82
Fruit juice, fresh (single or mixed)	51	315	96	0.30	160	296	482	14	334	95	0.28	312	71
Eggplant/entula sauce	48	194	60	0.31	95	182	325	13	152	69	0.45	136	77
Beef soup (broth only)	53	73	38	0.52	12	70	141	11	148	78	0.53	136	55*
Maize porridge, refined flour	55	540	227	0.42	293	547	1134	11	470	115	0.24	461	100

^a^PSDE, Portion size distribution estimation method; 24HR, 24 hour dietary recall, PCT, percentile.

^b^The percentage of portions for individual foods or mixed dishes with portion sizes (grams) falling within the 5^th^-95^th^ percentile range of portion sizes derived by the PSDE method.

* indicates statistically significant differences between portion sizes (grams) between the PSDE method and the 24HR survey data; Mann-Whitney U-Test, P < 0.05.

**Table 3:** Portion sizes (grams) for selected foods and composite dishes estimated from a PSDE survey and reported with low frequency (i.e., 4–6% of all food portions) in a 24HR survey in the same population^a^.

Data Source	PSDE Method							24HR Survey					
Food or Beverage	n	Mean	SD	CV	5th pct	Median	95th pct	n	Mean	SD	CV	Median	Portions within 5^th^-95^th^ pct (%)^b^
		grams							grams				
Individual food items													
Chips (French fried potatoes)	40	144	65	0.45	56	140	261	5	227	94	0.41	260	60
Groundnuts, roasted	50	51	24	0.47	20	48	100	5	67	35	0.52	67	67
Fish (large species), dried, boiled	49	104	40	0.38	43	93	196	5	27	25	0.93	21	20
Banana, small type	41	148	40	0.28	92	142	220	5	160	124	0.78	114	20
Samosa	49	64	63	0.98	12	61	191	5	95	45	0.47	94	100
Mixed dishes													
Plantain & beans (katogo)	48	463	154	0.33	260	440	764	6	434	136	0.31	432	100

^a^PSDE, Portion size distribution estimation method; 24HR, 24 hour dietary recall, PCT, percentile.

^b^The percentage of portions for individual foods or mixed dishes with portion sizes (grams) falling within the 5^th^-95^th^ percentile range of portion sizes derived by the PSDE method.

### 24HR survey

Only 18 individual food items and 11 mixed dishes had ≥10 reported portions consumed, including multiple portions consumed by the same person on the day of recall. Descriptive data (Tables 2 and 3) are not given for 4 individual food items as these were not considered in the portion size estimation activity; two had similar substitute foods included, one was to use a standard unit size as the basis for portion size estimation so was excluded and one food was not picked up in the food listing exercise. For those reported, the SDs were also relatively large and the CVs ranged from 0.24 to 0.93, with an average of 0.49.

### Comparison of portion sizes between the PSDE activity and 24HR survey

Of the foods reported with relatively high frequency in the 24HR survey (Table 2), the median portion sizes for many (15/25), but not all foods, were not significantly different from those determined in the PSDE method. For foods with medians that differed significantly, there was no systematic bias in the direction of difference. The percent of portion sizes reported in the 24HR survey that fell between the 5th and 95th percentiles determined by the PSDE method ranged from a low of 18% up to 100%. Of the foods reported with lower frequency in the 24HR survey (Table 3), results were similar. The percentage of portion sizes falling between the 5th and 95th percentiles differed markedly among the 6 food items shown here, ranging from 20 to 100%. The small sample of portion size data from the 24HR survey data and large variances for many foods precluded any statistical comparison between methods.

## Discussion

We have described two relatively low cost methods that could aid the development of semi-quantitative dietary assessment methods, as determined in a rural African population. A simple food and recipe listing method found that a scoring system was effective at identifying foods that were most likely to occur in a dietary survey. A relatively simple and rapid method to obtain distributions of portion sizes from a minimum sample indicated that for many foods, portion size distributions compared well with those obtained from standard 24HR methods, while several others did not.

The food listing method field-tested here provides a useful, scoring method to identify foods that should be included in dietary surveys using closed food lists, such as FFQ and SQ-FFQ methods. The foods scored as having a high or medium likelihood of being consumed covered the vast majority (i.e., 95%) of the total kilocalorie intake in the 24HR survey. This is important as it is a key criteria for developing adequate FFQ/SQ-FFQ methods [5]. This scoring process may also help to limit the number of foods included in a FFQ/SQ-FFQ tool and hence minimize respondent burden whilst still capturing the majority of nutritionally significant dietary intakes. Only a small number of food items (n = 5) occurred in the 24HR survey that were not included in the final food list. These were primarily low frequency foods, except for one food, sugarcane, that was reported by a large percentage (i.e., 23%) of individuals. Although this food listing process may be best suited to general surveys that aim to assess intakes from all foods, it could easily be adapted for use with specific food groups or foods providing specific nutrients.

In addition to use in FFQ and SQ-FFQ surveys, this food listing process could be used to support SQ-24HR methods such as those applying food photo atlases for portion size estimation (6,7). It would also be recommended to prepare for standard 24HR surveys as it allows survey designers to create prompt lists for relevant food details that should be probed for in an interview. This is expected to enhance the training and preparation of enumerators, and possibly the quality of data collected. Very little detail or specific guidance has been provided in the literature where such food listing processes are mentioned [6,9,10,19] or recommended [20].

The survey participant group interviews were more relevant to the food listing process as they focused on foods actually consumed in households, rather than on availability in the community as for the KIs. The latter may explain why more foods were mentioned by KIs and foods were scored with greater likelihood of consumption than in the survey participant interviews. Nonetheless, the KIs did serve to develop a more complete and locally relevant list of foods for use as a probing guide. The usefulness of combining expert consultation with ethnicity-specific details derived from the target population has been previously recommended [21].

This food listing method would be improved by including separate likelihood scores for foods consumed in different forms, including individual foods consumed in raw or cooked forms, foods cooked with or without oil, or as ingredients in mixed dishes so that these can be distinguished for inclusion in the PSDE. A limitation of the food listing activity is that snacks and beverages, including several baked goods and some commercial beverages were not adequately probed for during the interviews. More careful listing and probing, particularly of processed foods or snacks that are primarily purchased outside the home, is needed, as these were missed under the standard food group headings. Since these were not scored for likelihood of consumption in the food listing interviews, it's possible that PSDE data were collected unnecessarily for some of these. For example, of the 7 foods added to the food list by the researchers, 2 were not reported as consumed in the 24HR survey.

We developed and field tested a novel method to derive portion size distributions for the purpose of developing low cost and simple portion size estimation tools, such as food photo atlases, for use in large-scale dietary surveys. The systematic nature of this method represents an improvement on those reported in the literature for similar use, as previous methods have been largely qualitative in nature, and/or the level of representativeness of the usual range of portion sizes consumed by the target population is questionable [6,9,10,12,14,16].

Portion size estimation tools, including those using photographs, should reflect the range of amounts of foods typically consumed in the study population. A study among children [8] suggested that using age appropriate portion size options greatly reduced error in portion size estimation using photo series when depicting portion sizes actually consumed by children (i.e., average of 7% error in weight estimation) compared to using the lower range of portion size photos derived for use with adults (i.e., 46% error) [22]. In an extensive review of FFQ methods [5], it was suggested that in the absence of existing survey data, researchers conduct small surveys to derive portion size data, such as by using 24HR or diet history surveys. However, in practice this may be impractical due to cost, time, and technical skill required and a very large survey may be required to obtain an adequate sample from which to derive reliable portion size estimates. Indeed, in the 24HR survey conducted in this study including 115 respondents, the majority of foods were reported <10 times. The PSDE method tested here overcame that limitation by quickly obtaining a large sample for each food included.

This study did not aim to validate the PSDE method against a gold standard method, and the comparisons to the 24HR survey must be interpreted cautiously. First, as noted above, the sample for portion size estimates of many foods from the 24HR survey is small, thus limiting the ability to make direct statistical comparisons between methods. Second, the possibility of selection bias exists as response rates were <80% for each activity and 14% of women who participated in the PSDE did not participate in the large dietary survey as planned and SES data were not obtained. Hence, a difference in the SES of those women that affects usual portion sizes for some foods cannot be ruled out. Third, the PSDE method was limited in that it relied on longer term recall of portion sizes for foods consumed more than a day or week ago, and hence estimates may be distorted by memory. If some responses reflected 'usual' portion sizes rather than the portion they last consumed, the width of distributions may be attenuated as fewer extremes might be reported. This might partially explain why at least some portion sizes reported in the 24HR for most foods were outside the 5th and 95th percentiles of the PSDE distributions. Fourth, the 24HR method used different portion size estimation tools, which included photos of small, medium and large items (e.g., vegetables or roots used as ingredients), and dry rice or play dough to estimate volumes, and each of these is then converted to edible portion amounts in grams using previously obtained conversion factors. Thus, some lack of conformity with the PSDE likely occurred due to the difference in methods and additional error that may be introduced by these conversions.

The significant portion size differences for some foods between methods was concerning. For those, there was no apparent bias towards any one 24HR portion size estimation tool being consistently associated with low conformity. However, some foods with significantly different distributions had two or more distinct sizes available, such as bread slices from small and large loaves, and small and large *mandazi* (fritters) and mango varieties. In the PSDE method, both mango types were available for selection and the estimated portion sizes were combined in the same distribution. In the 24HR survey, portion sizes were estimated from photos of 3 different mango sizes used in previous surveys and derived from other areas. It's possible that the latter did not adequately represent the sizes of mangos available and resulted in an estimation bias. In this case, it may be necessary to obtain separate likelihood scores for the different sizes available, and possibly to produce separate PSDE data for the two sizes.

The lower conformity between methods for standard unit size items (e.g., bread slices, chapatti, mandazi) supports that basing portions on unit size with options for multiples or fractions of those units is a better approach than using continuous portion weights as derived from the PSDE [8]. In the case of beef, the reasons for discrepancy are less clear. Although both methods accounted for bone waste, in the PSDE, participants could select pieces with or without bone depending on how they could best visualize the portion size consumed whereas 24HR participants were asked to estimate only the edible portion of any meat served, which could introduce some error. The possibility of selection bias should also be considered as the sample reporting beef consumption in the 24HR survey was small and they may not have represented amounts consumed by the larger population. These are issues that should be studied more directly in establishing PSDE methods and the way foods are presented in the PSDE method, or estimated in the 24HR, as relevant for a particular population.

In addition to supporting the development of FFQs, SQ-FFQ, and simplified 24HRs using food photo atlases for portion size estimation, the PSDE method may also find use in nutrition research and advocacy tools that use linear programming. These methods identify foods that provide, or could provide, sufficient energy or nutrients to meet dietary requirements of a target population and require portion size estimates as input. These include Optifood, primarily used to derive food-based recommendations for optimizing diets of infants and young children [23], and the Cost of the Diet tool, an advocacy tool for estimating the cost of a nutritionally adequate diet [24]. Studies using Optifood typically use 24HR surveys to obtain input data [25,26], while the Cost of the Diet tool does not currently employ a satisfactory method for obtaining usual portion size data on which the models are based; the PSDE method may provide an option to improve this tool.

## Conclusions

We have identified a gap in available, well-described methods to collect data for deriving food lists and portion size estimates for use in a wide range of dietary assessment methods where existing, suitable dietary intake data are not available. Validating these methods was beyond the scope of this study. This would require a large-scale quantitative dietary intake survey in the same population with a large enough sample reporting intakes for a wide range of foods. However, this preliminary evaluation of the methods described and field-tested here, employing qualitative, semi-quantitative and quantitative methods with representative sampling, is well beyond what is currently described in the published literature and we recommend that these methods be further tested and validated when opportunities arise, such as in preparation for a large-scale or national dietary surveys.

## Supporting information

S1 File Sample size calculation for the sample population in a study to compare two simplified dietary assessment survey methods and a reference method (standard multiple-pass 24-hour dietary recall). (DOCX)

S1 Fig Example data collection sheet and guide for food listing with groups of survey participants. (DOCX)

S2 Fig Example data collection sheet and guide for detailing recipes of mixed dishes with survey participant groups. We recommend replacing the second column in the second table to record a likelihood score for each mixed dish mentioned, rather than obtaining ingredient information.
(DOCX)

S1 Datasets All data used to generate data and analyses in the study. Data are derived from the food listing interviews, the portion size distribution estimation (PSDE) activity, and the 24-hour dietary recall (24HR) survey.
(XLSX)
