# Supplementary material for: Simple methods to obtain food listing and portion size distribution estimates for use in semi-quantitative dietary assessment methods
Source: PLoS One. 2019 Oct 18;14(10):e0217379. doi: 10.1371/journal.pone.0217379 (PMC6799923; doi:10.1371/journal.pone.0217379)
Supplement: S1 File — (DOCX) [file pone.0217379.s001.docx]

The main objective of the larger field study was to design and field test two modified dietary data collection methods and tools using: (i) a Simplified 24HR recall format, and; (ii) a semi-quantitative food frequency questionnaire format, both using fixed portion size options presented in a food photo atlas and standard recipes applied to mixed dishes.

The study was carried out in five main phases. In **Phase 0**, a household census was conducted in the selected study area to identify eligible participants for the study activities. In **Phase 1**, a food and recipe list for the study area was compiled to inform the next data collection activities and the final design of the standard and simplified survey tools. In **Phase 2**, quantitative input data collection was carried out (portion size estimates for the test methods and standard recipe data for the test and reference methods). In **Phase 3**, the final versions of the Simplified 24HR and SQ-FFQ methods were field-tested and the surveys implemented concurrent with the Standard 24HR. **Phase 4** represents the data processing, analysis and reporting phase.

A schematic overview of the study activities is given in the figure below.

The sample size calculation for this study was based on the two-way comparison of key outcomes between each test method and the reference method. This main sample served as a pool from which to draw smaller samples for the input data collection activities. The sample size was calculated on the basis of one of the main outcome indicators - the percentage of individuals with intakes <100% of the EAR for a nutrient and the ability to detect a difference in percentage predicted by the different methods. For this, a Chi-Square Test for dichotomous outcomes, with a Type I error of α = 0.05 and power of β = 0.80 was used. Calculating the sample size for a range of detectable differences at different proportions of the binomial outcome between methods, we would be able to detect differences in proportions between either test method and the Standard 24 hour dietary recall method of between 19 percentage points (P0 = 0.50 and P1 = 0.69) and 9 percentage points (P0 = 0.90 and P1=0.99) with a sample size of 110 per group.

A summary of detectable differences and sample sizes between dietary recall methods to estimate prevalence of nutrient intakes <100% of the EAR (Chi-square test) is given in the table below.

|  | **% women with nutrient intake <100% EAR** | | | | |  |
| --- | --- | --- | --- | --- | --- | --- |
| **Negative test difference** | | |  | **Positive test difference** | | |
| **Sample size^a^** | Detectable difference | Test method^b^ | Reference method | Test method^b^ | Detectable difference | **Sample size^a^** |
| **104** | -19 | 31 | 50 | 69 | +19 | **104** |
| **107** | -19 | 36 | 55 | 73 | +18 | **110** |
| **108** | -19 | 41 | 60 | 78 | +18 | **102** |
| **106** | -19 | 46 | 65 | 82 | +17 | **105** |
| **103** | -19 | 51 | 70 | 86 | +16 | **104** |
| **108** | -18 | 57 | 75 | 90 | +15 | **100** |
| **110** | -17 | 63 | 80 | 93 | +13 | **107** |
| **107** | -16 | 69 | 85 | 96 | +11 | **110** |
| **112** | -14 | 76 | 90 | 99 | +9 | **100** |
| **106** | -12 | 83 | 95 | - | - |  |
| **100** | -9 | 90 | 99 | - | - |  |

^a^The sample size is based on a Chi-Square test of dichotomous outcomes in two prospective, independent case-control groups with equal numbers in each group; α = 0.05 and β = 0.80. The null hypothesis is that the failure rate (i.e., prevalence of intakes <100% of EAR) is equivalent between the control (Standard 24-hour recall) and the case (Simplified method, either 24-hour recall or semi-quantitative food frequency).

^b^The test will be repeated as two, two-way comparisons: (1) Standard 24-hour recall *vs* Simplified 24-hour recall, and (2) Standard 24-hour recall *vs* Semi-quantitative food frequency.
