# Supplementary material for: Simple methods to obtain food listing and portion size distribution estimates for use in semi-quantitative dietary assessment methods
Source: PLoS One. 2019 Oct 18;14(10):e0217379. doi: 10.1371/journal.pone.0217379 (PMC6799923; doi:10.1371/journal.pone.0217379)
Supplement: S1 Fig — (DOCX) [file pone.0217379.s002.docx]

|  | **DATE**: ____May 18, 2017___________________ | | | | | |  | | | **GROUP NUMBER /PARISH:** ___1/Namayiba______________ | | | | | |
| --- | --- | --- | --- | --- | --- | --- | --- | --- | --- | --- | --- | --- | --- | --- | --- |
|  |  |  | |  |  | |  | |  | |  | |  | | |
|  | **FOOD GROUP**: _NUTS AND SEEDS____________ | | | | | | | | | | | | | | |
|  |  |  | |  | |  | |  | | | |  | |  |  |
| **Food name, variety, form or type** | | **Is this food item consumed in FRESH, DRIED, or FLOUR form?** | | | | **LIKELIHOOD** food item will be consumed in July 2017 | | What **processing** methods are carried out on this food before consumption or addition to recipe? | | | | What **cooking methods** are used if/when consumed as an individual food item? | | What **mixed dishes** are consumed with this food as the main ingredient? | **ADD ANY COMMENTS/CONTEXT** |
| Pre-fill based on KI data sheet, use extra sheets, confirm/probe for extra foods | | Insert 'Y' (yes) or 'N' (no) | | | | 1=High; 2=Moderate; 3=Low;  4=Not likely at all | | Example: Pounded, dehulled, soaked, fermented, etc. | | | | Example:  Raw, boiled, steamed, roasted, deep fried in oil, etc. | | Write the name of the dish or product |  |
| 1 | Groundnuts | Fresh | Y | | | 4 | | Roasted and pounded | | | | Boiled, steamed or roasted | |  |  |
|  |  | Dried | Y | | | 1 | |  | | | | Roasted, steamed | |  |  |
|  |  | Flour | Y | | | 1 | |  | | | |  | | Groundnut sauce |  |
| 2 | Kulekula nuts | Fresh | N | | | - | |  | | | |  | |  |  |
|  |  | Dried | Y | | | 3 | | Roasted and pounded | | | | Roasted | |  |  |
|  |  | Flour | Y | | | 3 | |  | | | |  | | Kulekula sauce |  |
| 4 | Pumpkin seeds | Fresh | Y | | | 3 | |  | | | | Boiled (with pumpkin) | |  |  |
|  |  | Dried | Y | | | 3 | |  | | | | Roasted, pan-fried | |  |  |
|  |  | Flour | Y | | | 4 | |  | | | |  | | Pumpkin seed sauce |  |
| 5 | Sesame seeds | Fresh | N | | | - | |  | | | |  | |  |  |
|  |  | Dried | Y | | | 4 | | Roasted and pounded | | | |  | |  |  |
|  |  | Flour | N | | | - | |  | | | |  | |  |  |
| 6 | Sunflower seeds | Fresh | N | | | - | |  | | | |  | |  |  |
|  |  | Dried | Y | | | 4 | |  | | | |  | |  |  |
|  |  | Flour | N | | | - | |  | | | |  | |  |  |

| **MIXED DISHES PREPARED WITH FOOD ITEM (Complete Recipe Sheet for each Mixed Dish mentioned)** | |
| --- | --- |
| **Name of Mixed Dish** | **Description of ingredients*** |
| Groundnut sauce | Groundnut flour, tomato, onion |
| Kulekula sauce | Kulekula nut flour, tomato, onion |
| Pumpkin seed sauce | Pumpkin seed flour, tomato, onion |
|  |  |
|  |  |
