# Supplementary material for: Simple methods to obtain food listing and portion size distribution estimates for use in semi-quantitative dietary assessment methods
Source: PLoS One. 2019 Oct 18;14(10):e0217379. doi: 10.1371/journal.pone.0217379 (PMC6799923; doi:10.1371/journal.pone.0217379)
Supplement: S2 Fig — We recommend replacing the second column in the second table to record a likelihood score for each mixed dish mentioned, rather than obtaining ingredient information. (DOCX) [file pone.0217379.s003.docx]

|  | **DATE**: ____May 18, 2017___________________ | | | | | | | |  | | | **GROUP NUMBER /PARISH:** ___1/Namayiba______________ | | | | | | | |
| --- | --- | --- | --- | --- | --- | --- | --- | --- | --- | --- | --- | --- | --- | --- | --- | --- | --- | --- | --- |
|  |  | |  | |  |  | | |  | |  | | |  | |  | | | |
|  | **FOOD GROUP**: _NUTS AND SEEDS____________ | | | | | | | | | | | | | | | | | | |
| **RECIPE MAIN INGREDIENT:** Groundnuts___________________________________________ | | | | | | | | | | | | | | | | | | | |
|  | | | | | | |  |  | |  | | |  | |  | |  | |  |
| **NAME OF MIXED DISH** | | | | **NAME OF INGREDIENT** | | | | | | **Is this a MAJOR or MINOR ingredient?** | | | **FREQUENCY IN DISH** | | Probe for states and form of the ingredient before addition to dish | | | | |
|  |  |  |  |  |  |  |  |  |  |  |  |  |  |  | Added fresh or dried | | Cooking state as added | | How processed |
| Write the local name or basic description of dish | | | | Ingredient name Please list all possible ingredients in the dish | | | | | | Major or minor | | | 1=Always; 2=Often;  3=Sometimes;  4=Rare | | Fresh, dried | | Example: Raw, boiled, steamed, roasted, fried | | Example: Pounded, sliced/diced, mashed |
| Groundnut sauce with vegetables | | | | Groundnut flour | | | | | | Major | | | 1 | | Dried | | Raw | | Pounded |
|  |  |  |  | Water | | | | | |  | | | 1 | |  | |  | |  |
|  |  |  |  | Tomato | | | | | | minor | | | 2 | | Fresh | | Raw | | Sliced |
|  |  |  |  | Onion | | | | | | minor | | | 2 | | Fresh | | Raw | | Sliced |
|  |  |  |  | Eggplant/Entula | | | | | | minor | | | 2 | | Fresh | | Raw | | Sliced |
|  |  |  |  | Green pepper | | | | | | minor | | | 3 | | Fresh | | Raw | | Sliced |
|  |  |  |  | Carrot | | | | | | minor | | | 3 | | Fresh | | Raw | | Sliced |
|  |  |  |  | Green leaves | | | | | | minor | | | 4 | | Fresh | | Raw | | Sliced |
|  | | | |  | | | |  | |  | | |  | |  | |  | |  |
| **List the most popular ingredient combinations** | | | | | | | | | | | | | | | | | | | |
| 1 | | Groundnut flour, tomato, onion | | | | | | | | | | | | | | | |  |  |
| 2 | | Groundnut flour, tomato, onion, eggplant/entula | | | | | | | | | | | | | | | |  |  |
| 3 | |  | | | | | | | | | | | | | | | |  |  |
| 4 | |  | | | | | | | | | | | | | | | |  |  |
